# Necroptosis is associated with low procaspase-8 and active RIPK1 and −3 in human glioma cells

**DOI:** 10.18632/oncoscience.89

**Published:** 2014-10-22

**Authors:** Sara Melo-Lima, Maria Celeste Lopes, Faustino Mollinedo

**Affiliations:** ^1^ Instituto de Biología Molecular y Celular del Cáncer, Centro de Investigación del Cáncer, CSIC-Universidad de Salamanca, Campus Miguel de Unamuno, Salamanca, Spain; ^2^ Centre for Neuroscience and Cell Biology, University of Coimbra, Coimbra, Portugal; ^3^ Faculty of Pharmacy, University of Coimbra, Coimbra, Portugal; ^4^ Instituto de Investigación Biomédica de Salamanca (IBSAL), Hospital Universitario de Salamanca, Salamanca, Spain

**Keywords:** necroptosis, necrostatin-1, RIPK1, RIPK3, glioma, edelfosine

## Abstract

Necroptosis is a regulated necrotic cell death that involves receptor-interacting protein kinases RIPK1 and RIPK3. Here, we report that edelfosine triggers a rapid and massive cell death in human glioblastoma cells with characteristics of necrosis. Only a minor proportion of edelfosine-treated cells underwent caspase-dependent apoptosis. Autophagy and a rapid influx of extracellular calcium into the cells had little impact on cell death. Levels of procaspase-8 were very low in necroptosis-prone glioma cells compared with the levels in other cancer cell types that underwent apoptosis upon edelfosine treatment. The RIPK1-dependent necroptosis inhibitors necrostatin-1 (Nec-1) and Nec-1s as well as siRNA-mediated silencing of RIPK3 inhibited edelfosine-induced necroptosis, resulting in increased caspase-dependent apoptosis in edelfosine-treated glioblastoma U118 cells. Inhibition of the RIPK3 substrate MLKL with necrosulfonamide also increased apoptosis in edelfosine-treated cells. These data support a major role for RIPK1 and RIPK3 in the induction of necrotic cell death and in the switch from necrosis to apoptosis following edelfosine treatment. These results indicate that the ether lipid edelfosine exerts a rapid necroptotic cell death in apoptosis-reluctant glioblastoma cells, suggesting that induction of necroptosis could constitute a new approach for glioblastoma therapy.

## INTRODUCTION

Malignant glioma or glioblastoma is the most common and the most aggressive malignant primary brain tumor with a very poor prognosis. The survival rate for this tumor is extremely low because of its aggressiveness, ranging from about 3-4 months without treatment to up to a median survival of about 12-15 months following current medical therapy [1, 2]. Treatment of glioblastoma is a combined approach, including surgery, radiotherapy and chemotherapy. Current recommendations are based on radiotherapy combined with the oral alkylating drug temozolomide, which is able to cross the blood-brain barrier and exhibits a favorable side effect profile [3-5]. However, glioblastomas almost invariably recur near their initial sites leading rapidly to death [5, 6]. The cell death pathways induced by the triazene compound temozolomide in malignant glioma cells remain to be fully elucidated, but several studies have shown the induction of G_2_/M arrest and autophagy [7-9].

A number of survival signaling pathways can be activated constitutively in glioma cells, thus rendering these cells resistant to conventional chemotherapies and proapoptotic insults [10, 11]. The unfavorable prognosis for glioblastoma patients is strongly correlated to the intrinsic apoptosis resistance of glioblastoma cells [12, 13]. On these grounds, induction of alternative types of cell death could be an option for the treatment of glioblastoma. Thus, autophagic cell death by pro-autophagic drugs has been recently considered as an alternative and emerging concept to trigger glioma cell death and to exploit caspase-independent programmed cell death pathways for the development of novel glioma therapies [12].

The ether phospholipid edelfosine (1-*O*-octadecyl-2-*O*-methyl-*rac*-glycero-3-phosphocholine, ET-18-OCH3) is the prototype molecule of a family of unnatural lipids, collectively known as synthetic alkylphospholipid analogs (APLs), which promotes apoptosis in a variety of tumor cells [14-21]. Edelfosine has been shown to be an effective *in vitro* and *in vivo* antitumor drug, which acts through the reorganization of membrane domains, termed lipid rafts, as well as through an endoplasmic reticulum stress response, leading to caspase- and mitochondria-mediated apoptosis in different hematological and solid tumor cells [22-28].

Here we report that edelfosine induces mainly necroptosis in the U118 (U-118 MG) glioblastoma cell line, used as a brain tumor cell line model, whereas apoptosis and autophagy are relatively minor responses. Edelfosine-induced necroptototic response is very rapid and potent, thus suggesting a putative therapeutic role for necroptosis in brain tumor therapy.

## RESULTS

### Edelfosine promotes rapid cell death in U118 human glioma cells

**Figure 1:**
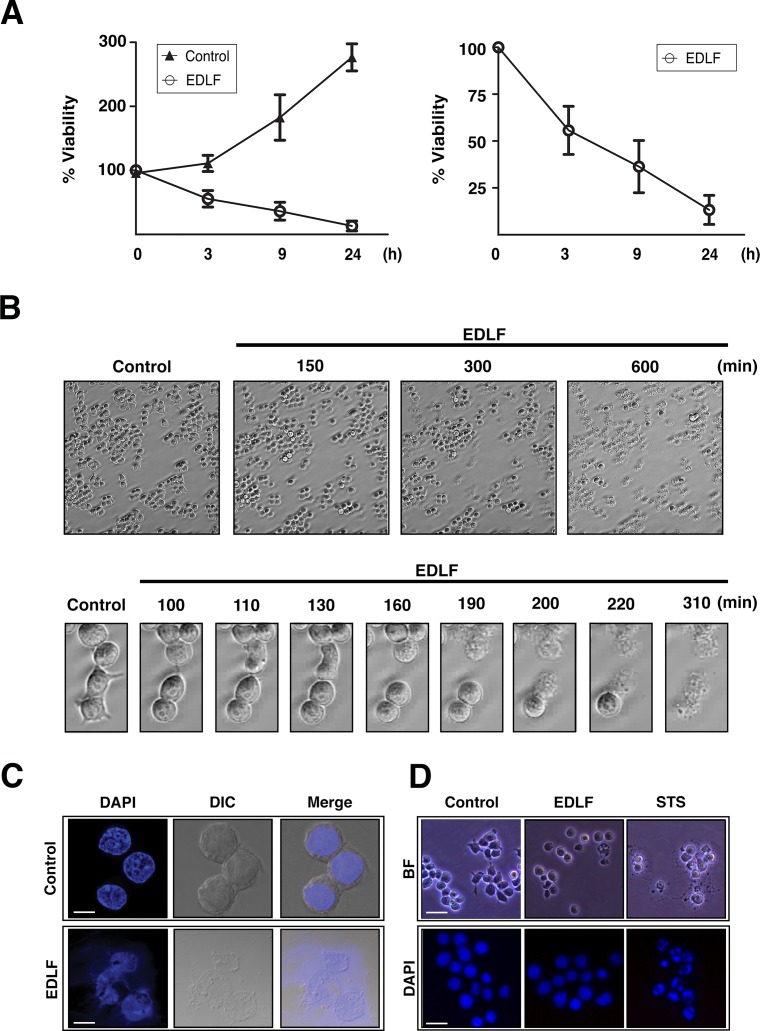
Edelfosine promotes rapid cell death in U118 human glioma cells (A) U118 cells were incubated in the absence (*Control*) or presence of 10 μM edelfosine (*EDLF*) for the indicated time points and then analyzed by MTT assay. Data are expressed as means ± SD of at least three independent experiments, each one performed in triplicate. The right plot shows only the measurements for edelfosine (*EDLF*)-treated cells for an easier appreciation of changes. (B) Selected phase-contrast time-lapse videomicroscopy frames (magnification, 10x) from U118 cells untreated (*Control*) or treated with 10 μM edelfosine (*EDLF*) at the indicated times. Lower panels show cells undergoing explosive death. The elapsed times in minutes are indicated on top of each frame. (C) Cell morphology of U118 cells untreated (*Control*) and treated with 10 μM edelfosine (*EDLF*) for 24 h. Differential interference contrast microscopy (*DIC*) and DAPI staining shows loss of plasma and nuclear membrane integrity after 24 h treatment. Magnification, 63x. *Bar*, 10 μm. (D) Brightfield microscopy (BF) and fluorescence microscopy photographs of DAPI-stained untreated control cells, cells treated with 10 μM edelfosine (*EDLF*) or with 0.5 μM staurosporine (*STS*) for 4 h. Chromatin condensation can be seen in staurosporine-treated cells, but not in edelfosine treated cells. Brightfield image magnification, 20x. DAPI image magnification, 40x. *Bar*, 40 μm (BF); *Bar*, 20 μm (DAPI).

Following MTT assays we found that incubation of the U118 human glioblastoma cell line with 10 μM edelfosine induced a rapid cell death response. U118 cells rapidly lost their ability to metabolize MTT following incubation with 10 μM edelfosine (Fig. 1A). Time-lapse videomicroscopy showed dramatic morphological changes as early as 150-180 min upon drug addition, showing apparently necrotic cell death, including cell swelling, membrane bubbling and plasma membrane disruption (Fig. 1B; Supplementary Videos S1 and S2). Most of the cells (~80%) showed morphologic features of necrosis after 24-h treatment (data not shown). Loss of nuclear membrane integrity was also readily detected by DAPI staining (Fig. 1C). In contrast, staurosporine-induced U118 cell death was accompanied by chromatin condensation, a typical hallmark of apoptosis, which was hardly observed following edelfosine treatment (Fig. 1D).

### Induction of apoptosis in edelfosine-treated U118 cells

**Figure 2:**
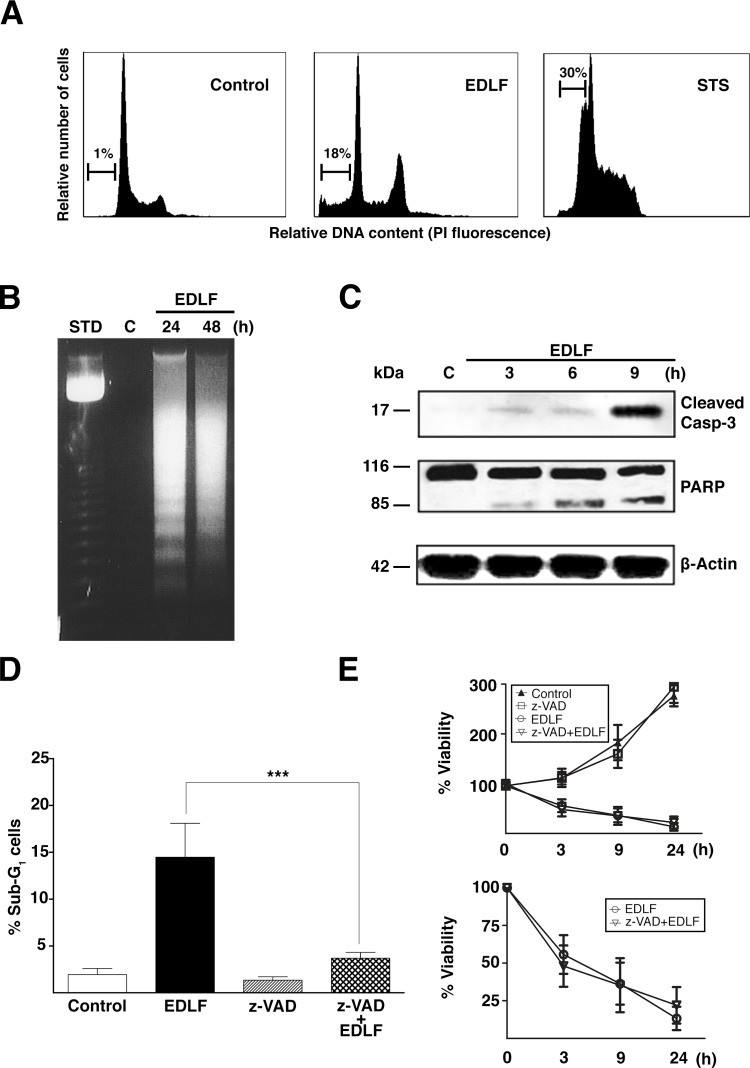
Edelfosine induces a minor apoptotic response in U118 cells (A) Representative cell cycle analysis histograms of untreated U118 cells (*Control*), U118 cells treated with 10 μM edelfosine (*EDLF*) for 24 h, and U118 cells treated with 0.5 μM staurosporine (*STS*) for 4 h. The percentage of apoptotic cells, identified as the sub-G_1_ population by flow cytometry, is indicated in each figure. (B) Cells treated with 10 μM edelfosine (*EDLF*) for 24 and 48 h were assayed for DNA fragmentation in agarose gels. Control untreated cells (*C*) were run in parallel in the same gels. A 123-bp DNA ladder was used as standard (*Std*). (C) Cells were untreated (Control, *C*) or treated with 10 μM edelfosine (*EDLF*) for the indicated times, and then analyzed by immunoblotting using specific antibodies against cleaved caspase-3 and PARP. Immunoblotting for β-actin was used as an internal control for equal protein loading in each lane. (D) Cells were preincubated without or with 100 μM pan-caspase inhibitor z-VAD-fmk (*z-VAD*) for 1 h, and then incubated in the absence (*Control*) or presence of 10 μM edelfosine (*EDLF*) for 24 h. Cells were analyzed by flow cytometry to evaluate apoptosis as the percentage of sub-G_1_ population in cell cycle analysis. Data shown are means ± SD of three independent experiments. ***, *P*<0.001 EDLF *vs*. z-VAD+EDLF, Student's *t* test. (E) MTT assays were conducted after culturing U118 cells without or with 100 μM pan-caspase inhibitor z-VAD-fmk (*z-VAD*) for 1 h, and then incubated in the absence (*Control*) or presence of 10 μM edelfosine (*EDLF*) at the indicated time points. Data are expressed as means ± SD of three independent experiments, each one performed in triplicate. The lower plot shows only the measurements for edelfosine- and z-VAD-fmk+edelfosine-treated cells for an easier appreciation of changes.

Because edelfosine has been reported to promote a potent and typical apoptosis in a wide number of tumor cells [15-17, 23, 29], we analyzed this cell death response in edelfosine-treated U118 cells. Only ~18% of the U118 cells treated with 10 μM edelfosine for 24 h displayed DNA degradation, as assessed by the percentage of cells in the sub-G_1_ region of cell cycle (Fig. 2A). This rather weak apoptotic response contrasted with the high DNA degradation detected following staurosporine treatment (Fig. 2A), used as a positive inducer for apoptosis [30]. Edelfosine treatment led to internucleosomal DNA degradation (Fig. 2B), a hallmark of apoptosis. In addition, edelfosine induced caspase-3 activation, as assessed by the appearance of the cleaved caspase-3 form, and the cleavage of poly(ADP-ribose) polymerase (PARP), a major caspase-3 substrate (Fig. 2C). Furthermore, preincubation with the pan-caspase inhibitor z-VAD-fmk completely blocked edelfosine-induced apoptosis (Fig. 2D), but was unable to inhibit the overall cell death response exerted by edelfosine in U118 cells (Fig. 2E). These results indicate that the minor edelfosine-induced caspase-dependent apoptosis response cannot account for the massive cell death detected in edelfosine-treated U118 cells.

### Induction of autophagy in edelfosine-treated U118 cells

**Figure 3:**
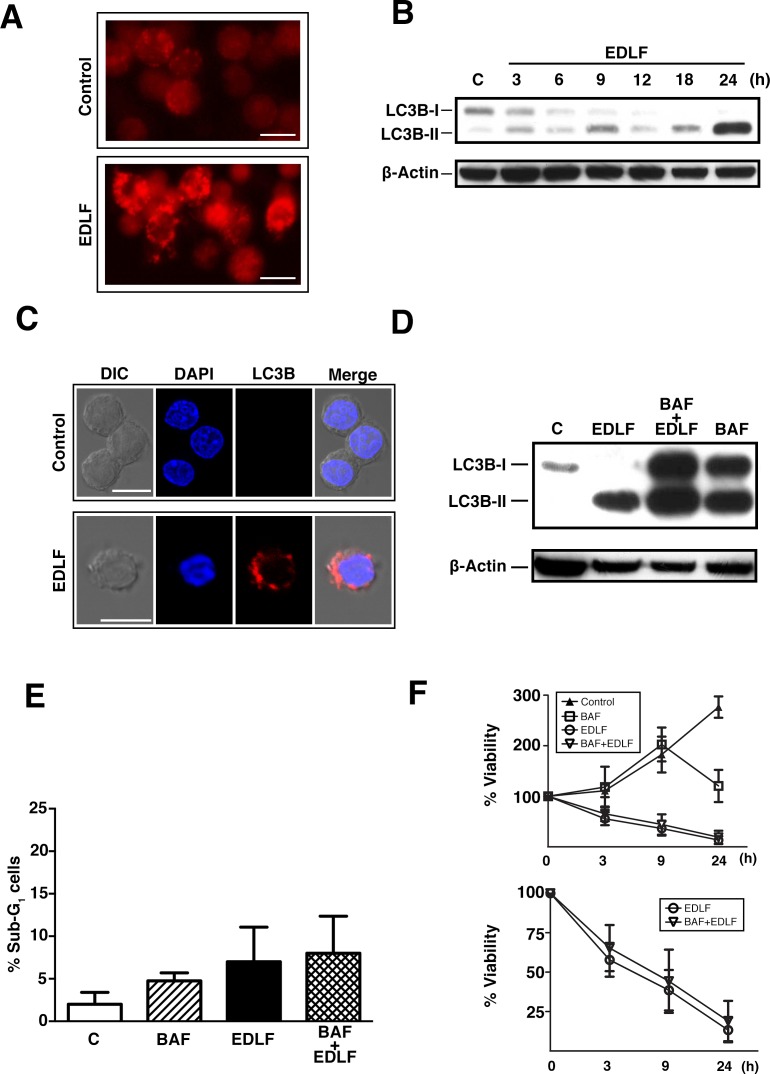
Induction of autophagy in edelfosine-treated U118 cells (A) Cells untreated (*Control*) and treated with 10 μM edelfosine (*EDLF*) for 24 h were stained with acridine orange and analyzed by fluorescence microscopy (magnification, 40x). (B) Western blot analysis of LC3B-I/II in cells untreated (Control, *C*) or treated with 10 μM edelfosine (*EDLF*) for the indicated times. Immunoblotting for β-actin was used as an internal control for equal protein loading in each lane. (C) Confocal immunofluorescence microscopy of LC3B punctae (red fluorescence) in U118 cells following treatment with 10 μM edelfosine (*EDLF*) for 3 h. Nuclei were labeled with DAPI (blue fluorescence). *Bar*, 10 μm. (D) Western blot analysis of LC3B-I/II in U118 cells treated with 10 μM edelfosine (*EDLF*), 25 nM bafilomycin A1 (*BAF*), or both (*BAF+EDLF*) for 24 h. β-Actin was used as a control for protein loading. (E) Cells were preincubated without or with 25 nM bafilomycin A1 (*BAF*) for 1 h, followed by incubation in the absence (Control, *C*) or presence of 10 μM edelfosine (*EDLF*) for 6 h, and then analyzed by flow cytometry to evaluate apoptosis. Data shown are means ± SD of three independent experiments. (F) MTT assay of U118 cells untreated (*Control*) or treated with 25 nM bafilomycin A1 (*BAF*) for 1 h, and then incubated in the absence or presence of 10 μM edelfosine (*EDLF*) at the indicated time points. Data are expressed as means ± SD of at least three independent experiments, each one performed in triplicate. The lower plot shows only the measurements for edelfosine- and bafilomycin A1+edelfosine-treated cells for an easier appreciation of changes.

The acidotropic agent acridine orange has been employed to monitor the development of acidic vesicular organelles (AVOs) during autophagy [31, 32]. We found an intense vital red fluorescence staining of U118 cells after edelfosine treatment (Fig. 3A), showing that edelfosine promotes the generation of acidic vacuoles in U118 cells. A major hallmark of autophagy lies in the conversion of microtubule-associated protein 1 light chain-3B (LC3B) from free form cytosolic LC3B-I (~18 kDa) to the phosphatidylethanolamine-conjugated LC3B-II form (~16 kDa), which is tightly bound to the membrane of the autophagosome [33]. Edelfosine treatment led to the rapid conversion of LC3B-I to LC3B-II after a 3-h treatment, reaching its maximum following 24-h treatment (Fig. 3B). The formation of LC3B-II, and thereby of autophagosomes, was evidenced by both Western blot (Fig. 3B) and confocal microscopy (Fig. 3C), this latter approach showing the fluorescent punctuate pattern of LC3B-II associated with autophagosomal membranes. To confirm that edelfosine was increasing the autophagic flux (autophagic vesicle formation and clearance by fusion with lysosomes), we used bafilomycin A1, which blocks fusion between lysosomes and autophagosomes and therefore leads to autophagosome accumulation. We observed an increase in the level of the LC3B-II form in cells treated with bafilomycin A1 in comparison with untreated control cells as well as with those treated with edelfosine alone (Fig. 3D), indicating ongoing autophagic flux and the blockade of the fusion between autophagosomes and lysosomes. This inhibition of the late stages of autophagy scarcely increased the apoptotic response (Fig. 3E), and did not increase overall viability upon edelfosine incubation, with no significant change in MTT reduction (Fig. 3F) or propidium iodide (PI) incorporation (data not shown). In addition, pretreatment of U118 cells with additional autophagy inhibitors, including 20 nM chloroquine or 200 nM wortmannin did not affect edelfosine-induced cell death response (data not shown).

### Edelfosine induces mainly necrotic cell death in U118 cells

**Figure 4:**
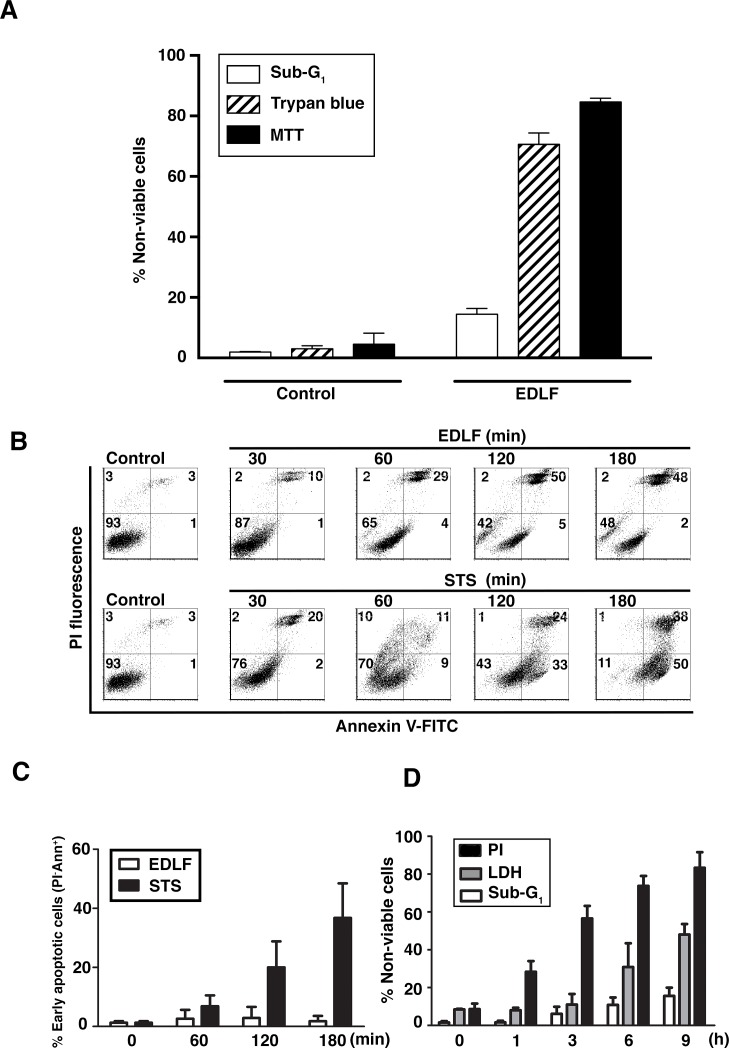
Loss of plasma membrane integrity in edelfosine-treated U118 cells (A) Percentage of non-viable cells in cells untreated (*Control*) or treated with 10 μM edelfosine (*EDLF*) for 24 h, as determined by three different methods: cell cycle analysis (sub-G_1_ population measured by flow cytometry), Trypan blue method, and MTT assay (cells unable to metabolize MTT). (B) Annexin V/PI staining was analyzed from cells untreated (*Control*) and treated with 10 μM edelfosine (*EDLF*) or with 0.5 μM staurosporine (*STS*) at the indicated time points. *Lower right quadrant* shows annexin V+/PI^−^ cells (early apoptotic cells). *Upper right quadrant* represents annexin V+/PI+ cells (necrotic or late apoptotic cells). Percentages of cells in each quadrant are indicated. Results are representative of three independent experiments. (C) Quantification of early apoptotic cells (annexin V+/PI-cells) at the indicated time points, following 10 μM edelfosine (*EDLF*) and 0.5 μM staurosporine (*STS*) treatments. (D) Cells were incubated with 10 μM edelfosine (*EDLF*) for the indicated periods of time, and then non-viable cells were measured using PI incorporation in non-permeabilized cells (necrosis), LDH release assays, and cell cycle analysis (quantification of apoptotic/sub-G_1_ population). Data shown in A, C and D are means ± SD of three independent experiments.

The above results showed that edelfosine-induced cell death in U118 cells was not primarily mediated by apoptosis or autophagy. The morphological changes observed in Fig. 1 apparently corresponded to necrosis, and this edelfosine-induced necrotic cell death was further assessed by the high presence of non-viable cells measured by Trypan blue staining, leading to similar figures as those obtained by the MTT assay (Fig. 4A), whereas only a minor proportion of cells showed a sub-G_1_ DNA content (Fig. 4A). To exclude the hypothesis that a secondary necrosis following apoptosis was occurring, we carried out time-course experiments using annexin V/PI assays. An increasing percentage of cells stained positive for both annexin V and PI following edelfosine treatment (Fig. 4B), whereas the apoptosis-inducer staurosporine [30] prompted a high percentage of annexin V+/PI-cells (Fig. 4B). After a 24-h treatment, most of the edelfosine-treated cells were annexin V+/PI+ (Supplementary Fig. S1). In contrast to edelfosine, which hardly induced apoptosis at early incubation times (Fig. 4C), staurosporine induced a high apoptotic response, as assessed by an increase in the annexin V+/PI-cell population (Fig. 4C) and by an increase in the hypodiploid sub-G_1_ cell population by cell cycle analysis (Fig. 2A). Plasma membrane permeability was also confirmed by the release of soluble cytosolic LDH into the culture medium at early incubation times following edelfosine addition, although LDH release showed a slower kinetics than that of PI incorporation in non-permeabilized cells (Fig. 4D). A weak increase in hypodiploid sub-G_1_ phase (apoptosis) was detected at a slower rate, after 6-9 h of treatment (Fig. 4D). Taking together, these data indicate a loss of cellular membrane integrity, a hallmark of necrotic cell death, and the onset of this type of cell death was detected long before the onset of apoptosis in edelfosine-treated cells. Thus, the necrotic response induced by edelfosine in U118 cells was a rapid and direct one, and not a consequence of secondary necrosis that usually takes place in the late phases of apoptosis.

### Necroptosis inhibitor necrostatin-1 significantly increases cellular viability after edelfosine treatment

**Figure 5:**
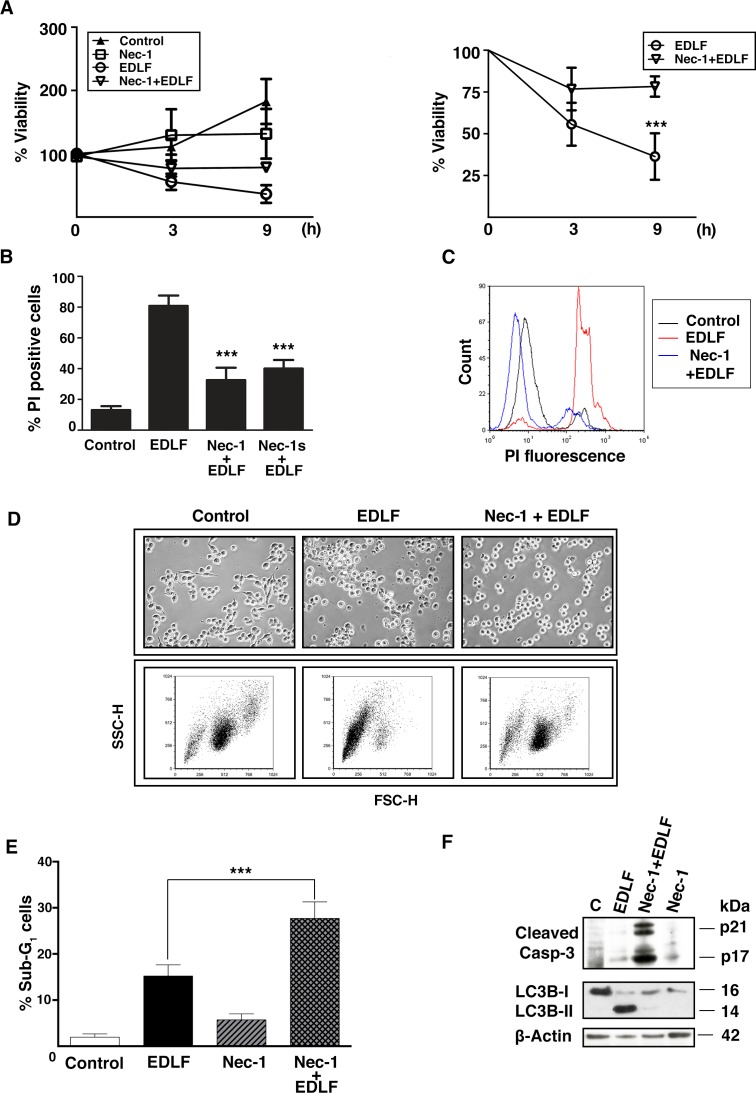
Induction of Nec-1-inhibitable necrosis in edelfosine-treated U118 cells (A) Cell proliferation was measured by MTT assay at the indicated time points, after culturing U118 cells without or with 200 μM Nec-1 (*Nec-1*) for 2 h, and then incubated in the absence or presence of 10 μM edelfosine (*EDLF*). Untreated control cells were run in parallel. Data are expressed as means ± SD of at least three independent experiments, each one performed in triplicate. The right plot shows only the measurements for edelfosine- and Nec-1+edelfosine-treated cells for an easier appreciation of changes. ***, *P*<0.001 EDLF *vs*. Nec-1+EDLF, Student's *t* test. (B) Quantification of U118 cells stained with PI after treatment with 10 μM edelfosine (*EDLF*) for 4 h without and with a pretreatment of 200 μM Nec-1 (*Nec-1+EDLF*) or 200 μM Nec-1s (*Nec-1s+EDLF*). Data shown are means ± SD of three independent experiments. ***, *P*<0.001 Nec-1+EDLF *vs.* EDLF; ***, *P*<0.001 Nec-1s+EDLF *vs.* EDLF, Student's *t* test. (C) Representative flow cytometry analysis histograms of PI incorporation showing: untretated control cells (*Control*), 10 μM edelfosine-treated cells (*EDLF*), and cells treated with Nec-1 (200 μM, 2 h pretreatment) + EDLF (10 μM) (*Nec-1+EDLF*) for 4 h. (D, *upper pannel*) Bright-field microscopy of untreated control cells, 10 μM edelfosine treated cells for 4 h (*EDLF*), and cells preincubated with 200 μM NEC-1 for 2 h and then treated for additional 4 h with 10 μM edelfosine (*Nec-1+EDLF*). Magnification, 20x. (D, *lower pannel*) FSC/SSC histograms of the cells treated as in the upper panels, showing cellular size (FSC-H) and granularity (SSC-H). Dead cells show lower FSC than living cells. (E) Cells were preincubated without or with 200 μM Nec-1 (*Nec-1*) for 2 h, then incubated in the absence or presence of 10 μM edelfosine (*EDLF*) for 24 h, and analyzed by flow cytometry to evaluate apoptosis. Untreated control cells were run in parallel. Data shown are means ± SD of three independent experiments. ***, *P*<0.001 EDLF *vs*. Nec-1+EDLF, Student's *t* test. (F) Cells were untreated (Control, *C*), treated with 10 μM edelfosine for 24 h (*EDLF*), pretreated with 200 μM Nec-1 for 2 h and then incubated with edelfosine for 24 h (*Nec-1+EDLF*), or treated with 200 μM Nec-1 for 26 h (2 h + 24 h). Cells were then analyzed by immunoblotting using specific antibodies against cleaved caspase-3 and LC3B-I/II. Immunoblotting for β-actin was used as an internal control for equal protein loading in each lane. Data shown in C, D and F are representative of three independent experiments.

We next examined whether the necrotic cell death process induced by edelfosine in U118 cells was a modulated one. Necroptosis is a mechanism of regulated necrosis that is dependent of receptor-interacting protein kinase-1 (RIPK1) and RIPK3 [34-36], and is inhibited by necrostatin-1 (Nec-1), a specific inhibitor of RIPK1 [37, 38]. Here, we found that Nec-1 was able to improve overall viability of edelfosine-treated U118 cells, as assessed by protecting MTT metabolism (Fig. 5A), reducing PI incorporation in non-permeabilized cells (Fig. 5B and C), and preventing necrotic morphology evaluated by either bright field microscopy (Fig. 5D, *upper*) or SSC/FSC flow cytometry analysis (Fig. 5D, *lower*). Preincubation of Nec-1 also induced a slight, but significant, increase in apoptosis in edelfosine-treated cells, as assessed by an increase in the level of sub-G_1_ DNA cell population (Fig. 5E) and caspase-3 activation (Fig. 5F). In addition, Nec-1 preincubation inhibited edelfosine-induced autophagy (Fig. 5F), suggesting that autophagy is a side-effect of the stress imposed to the U118 cells undergoing necrosis. Because Nec-1 is also known to inhibit indoleamine-2,3-dioxygenase (IDO) [39-41], we tested the effect of Nec-1s, a more specific RIPK1 inhibitor lacking the IDO-targeting effect [38, 40]. Nec-1s showed a similar protective effect to that observed with Nec-1, highly reducing PI incorporation in edelfosine-treated U118 cells (Fig. 5B), and thereby the effect of Nec-1 on PI uptake was not due to suppression of IDO activity.

### U118 cells express RIPK1 and have low levels of procaspase-8 and extrinsic apoptotic pathway molecules

**Figure 6:**
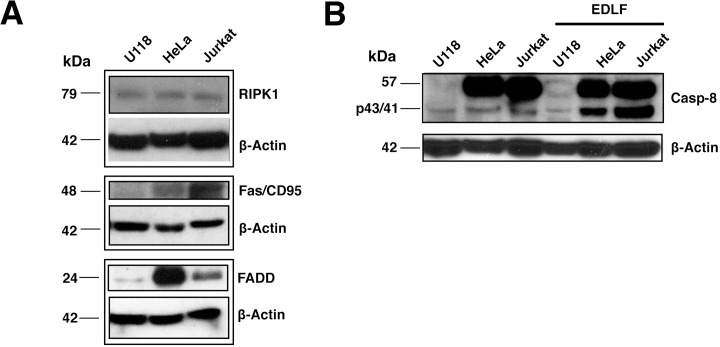
Relative protein levels of RIPK1, Fas/CD95, FADD and procaspase-8 in U118, HeLa and Jurkat cells (A) U118, HeLa and Jurkat cell lines were analyzed by immunoblotting using specific antibodies against RIPK1, Fas/CD95 and FADD. Immunoblotting for β-actin was used as an internal control for equal protein loading in each lane. (B) U118, HeLa and Jurkat cells untread and treated with 10 μM edelfosine (*EDLF*) for 24 h were analyzed by immunoblotting using a specific antibody that recognizes full-length 57-kDa procaspase-8 and p43/41 cleaved active caspase-8 fragments. The molecular weight of each immunodetected band is indicated. Data shown are representative of three experiments.

Edelfosine has been reported to induce apoptosis in a variety of hematological cancer cells through the recruitment into lipid rafts of extrinsic apoptotic pathway molecules forming clusters of Fas/CD95 death receptor and downstream signaling molecules, including FADD and procaspase-8 [22, 23, 28, 42, 43]. Here we found that U118 expressed RIPK1, a major protein in necroptosis, but very low levels of Fas/CD95, FADD and 57-kDa procaspase-8, critical proteins involved in the extrinsic pathway of apoptosis, as compared to HeLa (human cervical carcinoma) and Jurkat (human acute T-cell leukemia) cells (Fig. 6A and B), two cancer cell lines that readily undergo apoptosis following edelfosine treatment [22, 44]. In addition, as shown in Fig. 6B, a high level of caspase-8 activation, assessed by the generation of its 43/41-kDa cleaved form, was detected in both HeLa and Jurkat cells upon edelfosine treatment, but not in U118 cells. These data support the notion that low levels of procaspase-8 or lack of caspase-8 activation, together with a high RIPK1/procaspase-8 ratio, might favor the induction of necroptotic cell death in U118 cells.

### Involvement of RIPK3 in edelfosine-mediated necroptosis in U118 cells

**Figure 7:**
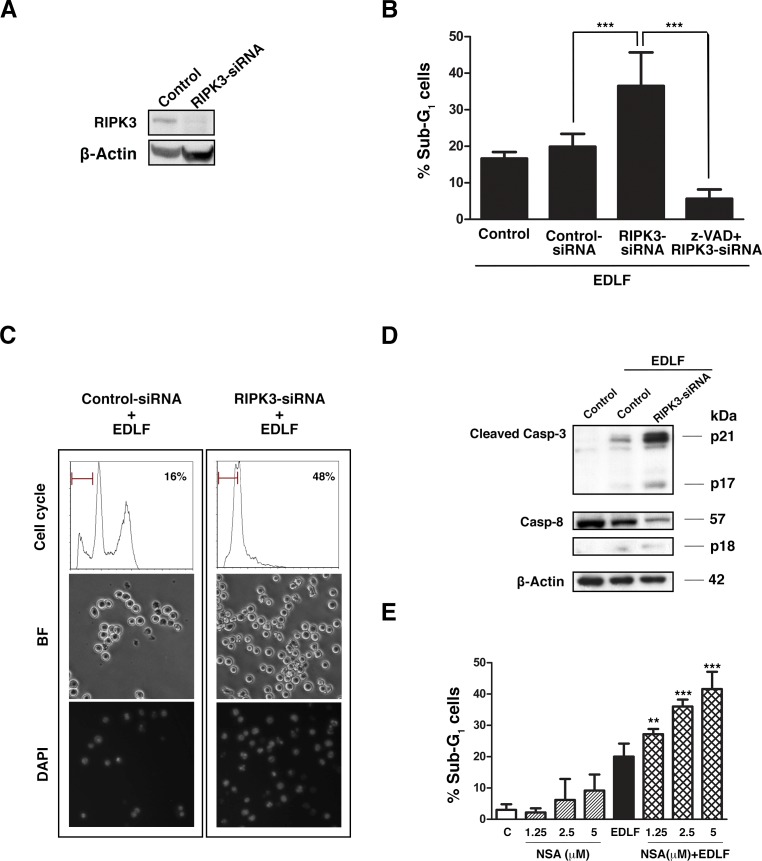
Effect of RIPK3 silencing on edelfosine-induced cell death in U118 cells (A) Immunoblot to assess knockdown of RIPK3 protein following transfection with RIPK3-siRNA (100 nM). β-Actin was used as a control for protein loading. Protein expression was evaluated 5 days after transfection. (B) U118 untreated control cells, cells transfected with a non-targeting sequence (siRNA Control), and cells transfected with 100 nM RIPK3-siRNA were incubated with 10 μM edelfosine for 20 h in the absence or presence of z-VAD-fmk, and apoptosis was evaluated by flow cytometry analysis of the cell cycle (sub-G_1_ cell population). Data shown are means ± SD of three independent experiments. ***, *P*<0.001 RIPK3-siRNA+EDLF *vs.* Control-siRNA+EDLF; ***, *P*<0.001 z-VAD+RIPK3-siRNA+EDLF *vs.* RIPK3-siRNA+EDLF, Student's *t* test. (C) Non-targeting siRNA (control)- and RIPK3-siRNA-transfected cells treated with 10 μM edelfosine were analyzed by cell cycle flow cytometry (sub-G_1_ population and percentages of sub-G_1_ cells are indicated in each histogram) after 20 h drug treatment (*upper panel*); brightfield microscopy to visualize cell morphology after 4 h edelfosine treatment (*middle panel*), and DAPI staining of nuclei following 20 h drug treatment (*lower panel*). (D) Immunoblotting for cleaved caspase-3 forms and caspase-8 (procaspase-8 and cleaved p18 fragment) in untreated cells (*Control*), and in control cells, and RIPK3-siRNA-transfected cells treated with edelfosine 10 μM for 9 h. β-Actin was used as a control for protein loading. (E) U118 cells were untreated (*C*) or treated with necrosulfonamide (NSA) at the indicated concentrations for 21 h, treated with 10 μM edelfosine for 20 h, and pretreated with NSA for 1 h and then treated with edelfosine for 20 h. Then apoptosis was evaluated by flow cytometry analysis of the cell cycle (sub-G_1_ cell population). Data shown are means ± SD of three independent experiments. **, *P*<0.01 and ***, *P*<0.001 NSA+EDLF *vs.* EDLF, Student's *t* test.

Necroptosis has been shown to depend on the activation of RIPK1 and RIPK3, which associate forming complexes that are referred to as necrosomes [45], RIPK3 being identified as a crucial regulator of necroptosis [46, 47]. We found that U118 cells expressed RIPK3, and RIPK3 silencing by using siRNA (Fig. 7A), dramatically reduced (~80%) necrotic phenotype and, similarly to what was observed by using necrostatin-1, induced apoptotic cell death following edelfosine treatment, as assessed by an increase in sub-G_1_ cell population through cell cycle analysis, and by the presence of morphologic features of apoptosis, including cell surface blebbing and chromatin condensation in DAPI-stained nuclei in edelfosine-treated U118 cells (Fig. 7B and C). This induction of apoptosis was further supported by caspase-3 and caspase-8 activation (Fig. 7D) and its total inhibition by the pan-caspase inhibitor z-VAD-fmk (Fig. 7B). Cells transfected with a non-targeting sequence behaved as untreated control cells (Fig. 7B). The mixed lineage kinase domain-like protein (MLKL) has been identified as a key mediator of necrosis signaling downstream of RIPK3 and the small molecule called (E)-N-(4-(N-(3-methoxypyrazin-2-yl) sulfamoyl)phenyl)-3-(5-nitrothiophene-2-yl)acrylamide, usually referred to as necrosulfonamide, blocks necrosis downstream of RIPK3 by covalently modifying MLKL [48]. Here, we found that necrosulfonamide also increased the induction of apoptosis in edelfosine-treated U118 cells (Fig. 7E).

### Edelfosine-induced U118 necroptotic cell death is independent of changes in intracellular calcium concentration

**Figure 8:**
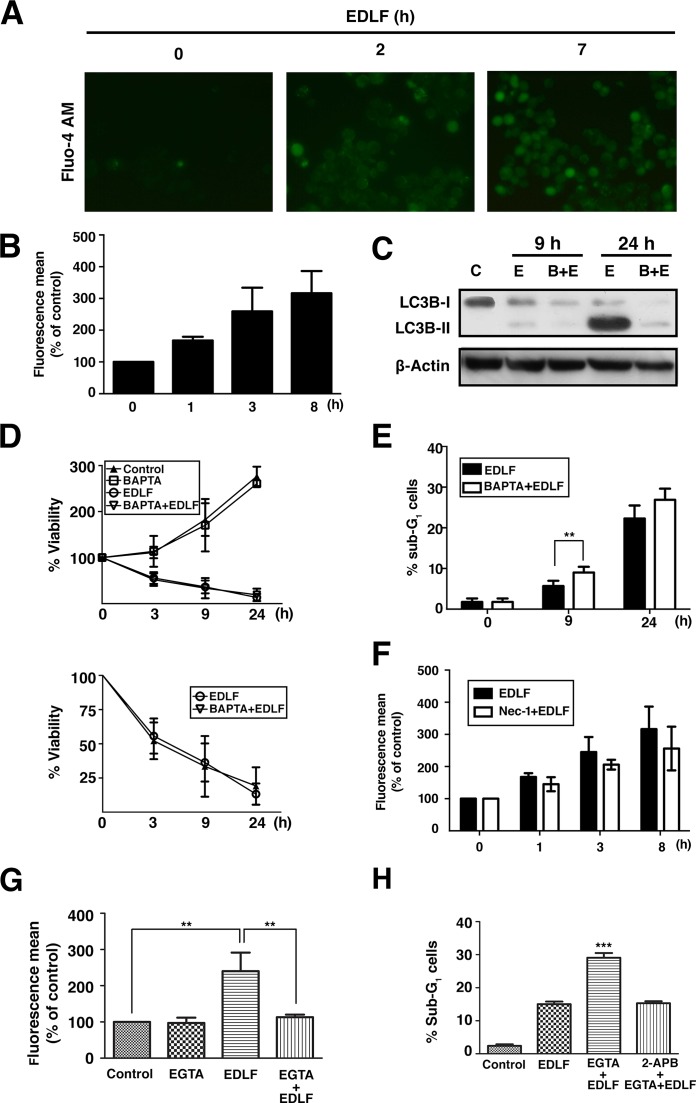
Changes in intracellular free calcium concentration in edelfosine-treated U118 cells (A) Cells were treated with 10 μM edelfosine (*EDLF*) for the indicated times followed by incubation with Fluo-4-AM for 30 min at 37°C, before being analyzed by fluorescence microscopy. (B) Cells were treated with 10 μM edelfosine (*EDLF*) for the indicated times, then incubated with Fluo-4-AM dye for 30 min and fluorescence intensity was measured by flow cytometry. (C) Untreated control cells (*C*), cells treated with 10 μM edelfosine (*E*) for the indicated times, or cells incubated with 4 μM BAPTA for 1 h and then treated with 10 μM edelfosine (*B+E*) for the indicated times, were analyzed by immunoblotting using a specific antibody for LC3B-I/II. (D) MTT assays were conducted after culturing U118 cells without or with 4 μM BAPTA-AM for 1 h, and then incubated in the absence or presence of 10 μM edelfosine (*EDLF*) at the indicated time points. Untreated control cells were run in parallel. Data shown are means ± SD of three independent experiments, each one performed in triplicate. The lower plot shows only the measurements for edelfosine- and BAPTA-AM+edelfosine-treated cells for an easier appreciation of changes. (E) Cells were preincubated without or with 4 μM BAPTA-AM for 1 h, and then treated with 10 μM edelfosine (*EDLF*) for the indicated time points and analyzed by flow cytometry to evaluate apoptosis. **, *P*<0.01 EDLF *vs*. BAPTA+EDLF, Student's *t* test. (F) Cells treated with 10 μM edelfosine for the indicated times without (*EDLF*) or with Nec-1 pretreatment (*Nec-1+EDLF*) were incubated with Fluo-4-AM for 30 min at 37°C, and fluorescence was measured by flow cytometry. (G) Cells incubated for 1 h without or with 10 mM EGTA, and then in the absence or presence of 10 μM edelfosine (*EDLF*) for 4 h, were incubated with Fluo-4-AM for 30 min and fluorescence was measured by flow cytometry. Untreated control cells were run in parallel. Data are expressed as means ± SD of three independent experiments. **, *P*<0.01, Student's *t* test. (H) Cells were pretreated with EGTA (10 mM) or 2-APB (60 μM) + EGTA (10 mM) for 1 h, and then incubated in the absence or presence of 10 μM edelfosine (*EDLF*) for 24 h, and analyzed by flow cytometry to evaluate apoptosis. Untreated control cells were run in parallel. Data are expressed as means ± SD of three independent experiments. ***, *P*<0.001 EGTA+EDLF *vs.* EDLF or 2-APB+EGTA+EDLF, Student's *t* test.

Because a connection between Ca2+ homeostasis and necrosis has been suggested [49, 50], we next examined whether calcium was involved in edelfosine-induced cell death by measuring intracellular calcium levels using the calcium indicator dye Fluo-4 AM. Incubation of U118 cells with edelfosine led to a rapid and persistent increase in the free intracellular calcium concentration (Fig. 8A and B). Following 24-h drug incubation, swollen dying cells still displayed bright green fluorescence, indicative of a high intracellular calcium concentration (data not shown). The membrane permeable calcium chelator BAPTA-AM, that inhibited ~55% the increase in free calcium concentration induced by edelfosine treatment, strongly diminished edelfosine-induced autophagy as assessed by a lower number of AVOs (data not shown) and a reduced conversion of LC3B-I to LC3B-II in drug-treated U118 cells (Fig. 8C). However, BAPTA-AM preincubation did not affect the overall cell survival measured by MTT assay (Fig. 8D), but slightly increased the apoptotic response, although the difference was only statistically significant at 9-h treatment (Fig. 8E). Furthermore, inhibition of necroptosis by Nec-1 prior to edelfosine treatment led to a lower increase in the intracellular calcium level, but this effect was not statistically significant (Fig. 8F). Preincubation with the extracellular calcium chelator EGTA dramatically diminished the level of intracellular calcium (Fig. 8G) and slightly potentiated edelfosine-induced apoptosis (Fig. 8H), this increased apoptotic response being blocked by the inhibitor of inositol 1,4,5-trisphosphate-mediated Ca2+ release 2-APB (2-aminoethoxydiphenyl borate) (Fig. 8H). Taken together, these data suggest that the increase in intracellular free calcium concentration induced by edelfosine is prompted mainly through an inward flux of extracellular calcium ions, a process that could be rather independent of, but concomitant with, the onset on necroptosis, and that is suggested to promote a rather antiapoptotic and proautophagic response. However, when the influx of extracellular free calcium is blocked, the previously reported release of intracellular free calcium from internal stores elicited by edelfosine in solid tumor cells [24, 27] seems to play a role in the triggering of the minor apoptotic cell death response triggered in U118 cells.

## DISCUSSION

The results reported here show for the first time that an APL molecule, edelfosine, is able to induce necroptosis in a tumor cell. The ether phospholipid edelfosine induces a rapid necrotic cell death in human U118 glioblastoma cells, which is inhibited by the specific RIPK1 inhibitors Nec-1 and Nec-1s as well as by RIPK3 silencing, and accounts for most of the cell death (~80%) occurring in edelfosine-treated U118 cells, thus involving both RIPK1 and RIPK3 in the cell death process. Our data also indicate that edelfosine elicits a minor caspase-dependent apoptotic response (~18%) in U118 cells. Necroptosis is a programmed and regulated necrosis process that is dependent on RIPK1 and RIPK3 [34, 35], and has been linked to death receptor activation [34, 51, 52]. Edelfosine has been found to promote apoptosis in a wide number of cancer cells through the involvement of Fas/CD95 death receptor [20, 22, 23, 53, 54], but the results reported here suggest that U118 glioma cells have some molecular features that make them prone to undergo this necroptotic cell death instead of apoptosis upon edelfosine incubation. In this regard, the relative ratio of RIPK1/procaspase-8 level was much higher in U118 cells than in HeLa and Jurkat cells, two cancer cell lines that readily undergo apoptosis following edelfosine treatment [22, 44], and might predispose U118 cells to undergo necroptosis upon edelfosine treatment. Our results agree with the notion that the mode of cell death is determined whether the cells are apoptosis-prone or apoptosis-reluctant, depending on the apoptotic machinery and levels of procaspases present in each specific cell type [55-57]. Thus, the cell's ability to undergo a certain type of cell death is mainly dictated by its inherent features of gene expression patterns related to cell death and/or by the putative activation of specific signaling pathways that modulate cell death. On these grounds, the data reported here suggest that U118 glioma cells are apoptosis-reluctant but necroptosis-prone, and their proneness to undergo necroptotic cell death is easily triggered by edelfosine. Interestingly, our results also show that necroptosis inhibition by RIPK1 inhibition and RIPK3 silencing causes U118 cells to undergo caspase-dependent apoptosis upon edelfosine treatment, thus turning the cell death response to caspase-dependent apoptosis when necroptosis is blocked.

Edelfosine has been previously reported to promote caspase-independent cell death in LN18 malignant glioma cell line [58]. Another APL, perifosine, has been reported to induce caspase-independent cell death in human prostate cancer PC-3 cells [59]. The APL miltefosine promotes cell-type-dependent apoptotic and non-apoptotic cell death processes in several human breast cancer cell lines [60]. However, necroptosis was not examined in the above studies.

Temozolomide, considered as the new gold standard for brain tumor therapy, has been reported to induce autophagy in malignant glioma cells [9]. Our data indicate that triggering of autophagy by edelfosine was not relevant for the induction of the major necroptotic cell death response. Suppression of autophagy promoted a slight increase of apoptosis, thus suggesting autophagy could work as a weak protective scenario for apoptosis in edelfosine-treated U118 cells. The potent necroptotic activity of edelfosine was exerted when this ether lipid was used at 10 μM, a concentration that fits well the drug plasma levels (10-20 μM) found in pharmacokinetic and *in vivo* studies [25, 26, 61]. Temozolomide at the rather high concentration, but clinically achievable, of 100 μM and after 72 h incubation induces autophagy, but not apoptosis in several malignant glioma cell lines [8]. On these grounds, the results reported here suggest that certain features of U118 glioma cells make them particularly sensitive to edelfosine.

We also found that edelfosine prompted an increase in free intracellular Ca2+ concentration in U118 cells that was mainly due to the entry of extracellular calcium. This increase in intracellular Ca2+ was not relevant to the induction of the necroptotic response, but seemed to be involved in the triggering of autophagy.

**Figure 9:**
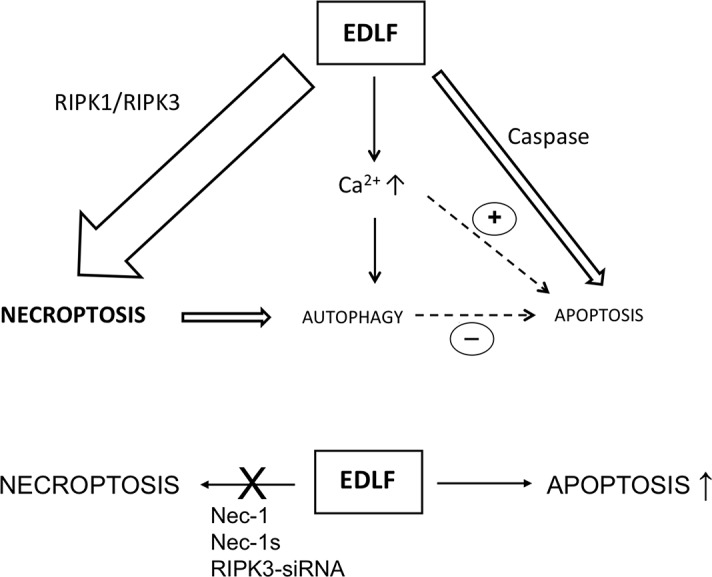
Schematic model for edelfosine-induced cell death in U118 cells (*upper*) This is a schematic diagram that depicts the cell death mechanisms triggered during the edelfosine killing action in U118 glioblastoma cells, and highlights the involvement of RIPK1/RPK3-mediated necroptotosis in the process. (*lower*) When necroptosis is inhibited, the caspase-dependent apoptotic response is potentiated following edelfosine treatment. See the text for details.

Figure 9 depicts a scheme for the relative participation of three major types of cell death in the demise of U118 cells upon edelfosine treatment. U118 cells treated with edelfosine primarily undergo necroptosis, involving RIPK1 and RIPK3 (Fig. 9, *upper*), and inhibition of this necrotic response results in an increase in caspase-dependent apoptosis (Fig. 9, *lower*), thus highlighting the potent pro-cell death signaling triggered by edelfosine in this glioma cell line. The fact that blockade of necroptosis led to increased apoptosis shows that edelfosine can activate both cell death pathways in U118 cells, and therefore it can be an effective drug for the demise of glioblastoma cells. This warrants further studies on the putative effects of this ether lipid in glioblastoma. The data reported here also suggest that U118 cells can constitute a useful cell model to elucidate the mechanisms that dictate the cellular decision to undergo alternative cell death pathways, such as apoptosis or necroptosis, as well as the processes mediating the triggering and execution of necroptosis. Our data also highlight the use of U118 glioma cells for the search of new therapeutic approaches to treat glioblastoma by promoting distinct types of cell death.

## METHODS

### Cell culture

Cells were grown at 37°C in DMEM (Gibco, Life Technologies Corporation) (U118 and HeLa) or RPMI-1640 (Gibco) (Jurkat) supplemented with 10% heat-inactivated FBS, 2 mM L-glutamine, 100 U/ml penicillin, 100 μg/ml streptomycin in a humidified atmosphere containing 5% CO2.

### Cell proliferation and viability assays

Cell proliferation and viability was assessed by the MTT (3-(4,5-dimethylthiazol-2-yl)-2,5-diphenyltetrazolium bromide) assay, through MTT conversion into colored formazan product by metabolically active cells [62]. Absorbance was measured using a spectrophotometric microplate reader with reference filter at 630 nm and reading filter at 570 nm. Each determination was performed in triplicate.

Cell viability was also evaluated by using Trypan Blue dye reagent. Non-viable cells stained blue, while viable cells excluded Trypan Blue and showed normal refringent cytoplasm. Samples were counted under a light microscope, and the percentage of non-viable cells was determined.

Cytotoxicity was also analyzed by lactate dehydrogenase (LDH) assay by using the Cytotoxicity Detection Kit (LDH) (Roche, Basel, Switzerland), according to the manufacturer's instructions. Absorbance was read in a spectrophotometric microplate reader at 450 nm. Values were normalized as percentages to untreated cells and shown as % viability relative to untreated cells.

Edelfosine was obtained from R. Berchtold (Biochemisches Labor, Bern, Switzerland). A stock solution was prepared at 2 mM in culture medium containing 10% (v/v) FBS by heating at 50°C for 30 min, as previously described [15].

### Measurement of apoptosis by flow cytometry

Quantitation of apoptotic cells was calculated as the percentage of cells in the sub-G_1_ region (hypodiploidy) following cell-cycle analysis as previously described [17].

### Analysis of DNA fragmentation in agarose gels

To assess apoptosis, we isolated fragmented DNA as previously described [14, 63], and internucleosomal DNA degradation was detected by electrophoretic analysis in 1% agarose gels (containing 0.5 μg/ml ethidium bromide) and UV transillumination.

### PI exclusion assay

This assay was used to evaluate plasma membrane integrity. Cells, resuspended in PBS containing 10 μg/ml PI, were incubated in the dark for 15 min at room temperature, and then analyzed by flow cytometry (590 nm) to determine the proportion of cells with increased permeability to PI (PI+-cells) as the percentage of cells with increased red fluorescence (strong shift in FL-2 values, *log* scale) with respect to the basal red fluorescence observed in untreated control cells.

### Annexin V/PI assay

Control and treated cells were collected, washed with PBS, and incubated in 100 μl annexin V binding buffer 1x (BD Pharmingen, San Jose, CA) containing 5 μl annexin V-FITC (BD Pharmingen) and 10 μg/ml PI for 15 min at room temperature. Samples were analyzed by flow cytometry with simultaneous monitoring of green fluorescence (530 nm, 30 nm band-pass filter) for annexin V-FITC and red fluorescence (long-pass emission filter that transmits light >650 nm) for PI.

### Supravital cell staining with acridine orange

Cells were incubated with 1 μg/ml acridine orange (Molecular Probes, Leiden, The Netherlands) for 15 min at 37°C, and then analyzed under a Nikon Eclipse Ti-S fluorescence microscope using an excitation filter of 550 nm (540-560 nm) and a long pass >610 nm emission/barrier filter.

### Measurement of intracellular calcium

Cells were incubated with 2 μM Fluo-4 AM dye (Molecular Probes) for 30 min at 37°C, and then analyzed by fluorescence microscopy or by flow cytometry.

### siRNA transfection

ON-TARGETplus SMART pool for human RIPK3 (L-003534-00) siRNA, which included a mixture of four specific siRNAs provided as a single reagent, as well as a non-targeting pool siRNA (D-001810-10-05) were purchased from Thermo Scientific (Pittsburgh, PA). U118 cells at a density of 8 × 104 cell per well in 6-well plates were transfected with 100 nM RIPK3-siRNA using siPORT NeoFX transfection agent (Life Technologies) according to the manufacturer's instructions. Protein knockdown was assessed between two and five days after transfection, and best knockdown rates were obtained following 5 days after transfection and used in the experiments shown in this work. Efficiency of RIPK3 knockdown was quantified with ImageJ software after Western blotting and using β-actin as protein control.

### Western blot analysis

Thirty to fifty micrograms of protein extracts from 4-5 × 106 cells, prepared as previously described [24] were subjected to SDS-polyacrylamide gels, transferred to Immobilon-P PVDF membranes (Merck Millipore, Darmstadt, Germany), blocked with 5% (w/v) defatted milk powder in TBST (50 mM Tris–HCl, pH 8.0, 150 mM NaCl, 0.1% Tween 20) for 1 h at room temperature, and incubated for 1 h at room temperature, or overnight at 4 °C, with the following specific antibodies: anti-17- and 19-kDa cleaved caspase-3 (Asp 175) rabbit polyclonal antibody (1:1000 dilution, Cell Signaling Technology); C2.10 anti-116 kDa PARP mouse monoclonal antibody, that also detects the 85 kDa cleavage product (1:1000 dilution, BD Biosciences); anti-18-kDa (LC3B-I)/16-kDa (LC3B-II) rabbit polyclonal antibody (1:1000 dilution, Cell Signaling Technology); anti-79-kDa RIPK1 rabbit polyclonal antibody (1:1000 dilution, Cell Signaling Technology); anti-62-kDa RIPK3 rabbit monoclonal antibody (1:1000 dilution, Cell Signaling Technology); anti-48-kDa Fas/CD95 rabbit polyclonal antibody (1:500 dilution, Santa Cruz Biotechnology Inc., Santa Cruz, CA); anti-24-kDa FADD mouse monoclonal antibody (1:1000 dilution, BD Transduction Laboratories); 1C12 anti-57 kDa procaspase-8 mouse monoclonal antibody, that also detects the cleaved p43/41 and p18 subunits (1:1000 dilution, Cell Signaling Technology). Secondary antibodies were anti-mouse or anti-rabbit immunoglobulins conjugated to horseradish peroxidase (GE Healthcare, Princeton, NJ). Signals were detected using an ECL kit (GE Healthcare).

### Time-lapse videomicroscopy

Cells were recorded by time-lapse microscopy using a Nikon Eclipse TE2000-E microscope that was enclosed in a Plexiglass box where cells were maintained under a humidified air of 5% CO2 at 37°C using OKO-Lab technology. MetaMorph software was used for image acquisition and processing. Frames were taken every 10 min for 9 h.

### Confocal microscopy

1 × 106 cells were seeded on 6-well plates, each well containing a sterile glass coverslip coated with poly-L-lysine. Untreated and edelfosine-treated cells were fixed in formaldehyde (4% in PBS) for 20 min at room temperature. After fixation, cells were permeabilized in cold PBS containing 0.1% Triton X-100 for 1 min and rinsed thoroughly with PBS. Staining was performed incubating the coverslip with a specific anti-LC3B rabbit polyclonal antibody (Cell Signaling Technology) (1:100 dilution) for 1-h at room temperature, followed by 1-h at room temperature with CY3-conjugated anti-rabbit immunoglobulin (Ig) antibody (diluted 1:150 in PBS; Jackson ImmunoResearch, West Grove, PA), and DAPI staining (0.5 μg/ml; 5 min). Each incubation was followed by 2 washes in PBS. Stained coverslips were then mounted on slides using the antifading reagent SlowFade Gold (Invitrogen, Eugene, OR) to preserve fluorescence signal intensity. Samples were analyzed by microscopy using a confocal Leica SP5 microscope and LAS AF software.

### Statistical analysis

Results are expressed as means ± SD of the number of experiments indicated. Comparisons between two experimental groups were determined using Student's *t*-test with *P*-value<0.05 indicating statistical significance.

## SUPPLEMENTARY MATERIAL FIGURE AND VIDEOS






