# Supplementary material for: Necroptosis is associated with low procaspase-8 and active RIPK1 and −3 in human glioma cells
Source: Oncoscience. 2014 Oct 22;1(10):649–64. doi: 10.18632/oncoscience.89 (PMC4278276; doi:10.18632/oncoscience.89)
Supplement: Supplementary file 1 [file oncoscience-01-0649-s001.pdf]

Necroptosis is associated with low procaspase-8 and active RIPK1 and -3 in human glioma cells

Supplementary Material

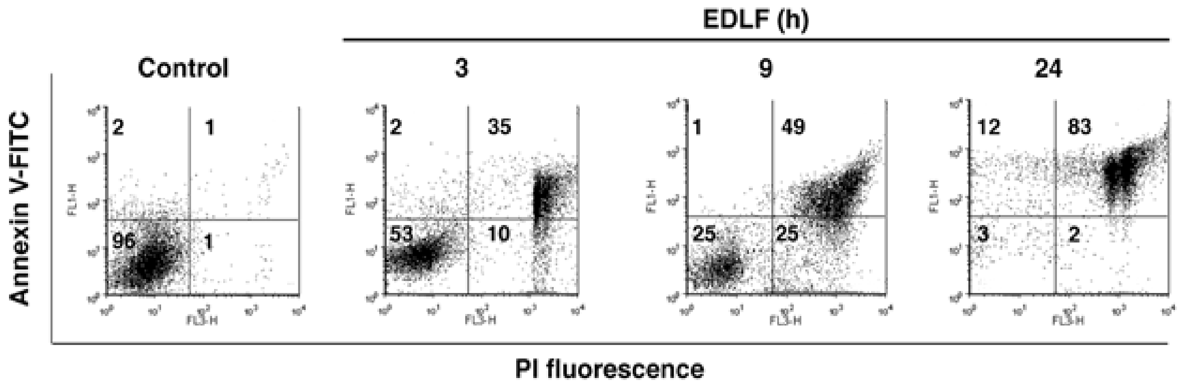

Supplementary Figure S1: Annexin V/PI staining in U118 cells treated with edelfosine for different incubation times. Annexin V/PI staining was analyzed from cells untreated (*Control*) and treated with 10 µM edelfosine (*EDLF*) at the indicated time points. *Upper left quadrant* shows annexin V^+/PI^- cells (early apoptotic cells). *Upper right quadrant* represents annexin V^+/PI^+ cells (necrotic or late apoptotic cells). Percentages of cells in each quadrant are indicated.

Supplementary Video S1: Time-lapse videomicroscopy of untreated control U118 cells. Cells were grown in complete DMEM culture medium supplemented with 10% heat-inactivated FBS. Recording time: 9 h. Magnification: 10x.

Supplementary Video S2: Time-lapse videomicroscopy of the effect of edelfosine on U118 cells. Film recording started immediately after addition of 10 µM edelfosine on U118 cells grown in complete DMEM culture medium supplemented with 10% heat-inactivated FBS. Recording time: 9 h. Magnification: 10x.
